# Metagenomic comparison of intestinal microbiota between normal and liver fibrotic rhesus macaques (*Macaca mulatta*)

**DOI:** 10.1038/s41598-024-64397-7

**Published:** 2024-07-08

**Authors:** Yuankui Wei, Junhui Li, Baoqiang Zhu, Qi Hu, Ming Lan, Jia Zhou, Jianbo Luo, Wanlong Zhu, Yong Lai, Enwu Long, Liang Zhou

**Affiliations:** 1 School of Pharmacy, Southwest Medical University, Luzhou, Sichuan China; 2 Departmemt of Institute of Laboratory Animal Sciences, Sichuan Provincial People’s Hospital, University of Electronic Science and Technology of China, Chengdu, Sichuan China; 3 Department of Pharmacy, 363 Hospital, Chengdu, Sichuan China; 4 Institute of Hospital of Chengdu University of Traditional Chinese Medicine, Chengdu, Sichuan China; 5 Department of Pharmacy, The Second People’s Hospital of Panzhihua, Panzhihua, Sichuan China; 6 Department of Pharmacy, Personalized Drug Therapy Key Laboratory of Sichuan Province, Sichuan Academy of Medical Sciences & Sichuan Provincial People’s Hospital, School of Medicine, University of Electronic Science and Technology of China, Chengdu, Sichuan China

**Keywords:** Rhesus macaques, *Macaca mulatta*, Liver fibrosis, Intestinal microbiota, 16S rRNA gene sequencing, Metagenomic, Microbiology, Zoology

## Abstract

Liver fibrosis is an important pathological process in chronic liver disease and cirrhosis. Recent studies have found a close association between intestinal microbiota and the development of liver fibrosis. To determine whether there are differences in the intestinal microbiota between rhesus macaques with liver fibrosis (MG) and normal rhesus macaques (MN), fecal samples were collected from 8 male MG and 12 male MN. The biological composition of the intestinal microbiota was then detected using 16S rRNA gene sequencing. The results revealed statistically significant differences in ASVs and Chao1 in the alpha-diversity and the beta-diversity of intestinal microbiota between MG and MN. Both groups shared *Prevotella* and *Lactobacillus* as common dominant microbiota. However, beneficial bacteria such as *Lactobacillus* were significantly less abundant in MG (*P* = 0.02). Predictive functional analysis using PICRUSt2 gene prediction revealed that MG exhibited a higher relative abundance of functions related to substance transport and metabolic pathways. This study may provide insight into further exploration of the mechanisms by which intestinal microbiota affect liver fibrosis and its potential future use in treating liver fibrosis.

## Introduction

The liver is a very important metabolic organ in the body, and various endogenous and exogenous factors may lead to abnormal liver function and liver diseases. When pathogenic factors cause changes in the microenvironment of hepatocytes, it results in an impact on the function of these cells^1^. Disruption of the balance of hepatic extracellular matrix and excessive production of extracellular matrix result in abnormal proliferation of hepatic connective tissue, known as fibrosis^2^. Liver fibrosis can easily progress to chronic liver disease, cirrhosis, or even liver cancer, causing significant harm to patients. Current studies^3^ have also shown that liver fibrosis is associated with various risk factors for cardiometabolic diseases. Currently, there have been no clear treatment methods for liver fibrosis. Therefore, animal disease models of liver fibrosis are of great significance in exploring the development process and treatment methods^4^. Non-human primate animal models of fibrosis, specifically rhesus macaques, are more valuable compared to mice, rabbits, and other small experimental animals. Rhesus macaques have physiological structures and functions that are more similar to those of human beings. Liver fibrosis models in macaques can substitute for humans in drug screening for liver fibrosis and provide the possibility of obtaining other models such as cirrhosis and hepatocellular carcinoma. Moreover, establishing a model of hereditary liver fibrosis would be a significant step towards the prevention and treatment of liver fibrosis in the future.

The intestinal microbiota refers to the microbial community that resides in the intestine. In recent years, there have been studies on the interaction between the intestinal microbiota and various diseases. One study^5^ showed that dysbiosis of the intestinal microbiota has detrimental health effects on the human body, leading to gastrointestinal diseases, cardiovascular diseases, metabolic disorders, and other diseases. Several studies^6–8^ have demonstrated a connection between the intestinal microbiota and the development of liver fibrosis. Another study^9^ used 16S rRNA gene sequencing to compare the gut microbiota of children and adolescents in three states: nonalcoholic steatohepatitis (NASH), obesity, and good health. The research discovered that each health condition was associated with a distinct type of intestinal microbiota, suggesting a strong correlation between the intestinal microbiota and liver health. Research has indicated that certain microbial-derived metabolites (such as trimethylamine, secondary bile acids, short-chain fatty acids, and ethanol) play a pathogenic role in the development of nonalcoholic fatty liver disease^10^. The gut-liver axis refers to the interaction between the gut, its microbiota, and the liver through the portal vein. Maintaining the balance of the microbial community is crucial for the homeostasis of the gut-liver axis. Disruption of the gut-liver axis can contribute to the progression of most chronic liver diseases. A study based on the gut-liver axis theory^11^ found that inhibiting gastric acid secretion through the use of proton-pump inhibitors (PPIs) can alter the composition of the intestinal microbiota and worsen alcohol-induced liver disease, leading to more severe levels of liver damage. Increased use of PPIs may be responsible for the rising incidence of chronic liver disease.

The rhesus macaque, a non-human primate, has long been regarded as an ideal animal model for research purposes. Although previous studies^12^ have reported on the intestinal microbiota of rhesus macaques, there is limited literature on the disparities between the intestinal microbiota of various disease models and healthy rhesus macaques^13–16^. Additionally, there has been currently a lack of data on the intestinal microbiota of rhesus macaques with liver fibrosis (MG) models, both domestically and internationally. In this study, we utilized 16S rRNA gene sequencing to investigate the differences in the intestinal microbiota between normal rhesus macaques (MN) and MG. We analyzed the difference of their composition and made functional predictions based on our findings.

## Results

The 16S rRNA gene sequencing was performed on the feces of 12 male MN and 8 male MG. Amplicon sequence variants (ASVs) are DNA sequences obtained from high-throughput marker gene analyses that have better specificity and lower false sequence rates compared to operational taxonomic units^17^. ASVs allowed for species identification and abundance determination through species annotation, as well as provided data for alpha-diversity and beta-diversity analyses. From the 12 MN, we obtained 1,251,927 effective reads (104,327 reads per sample), corresponding to a total of 1,850 ASVs. For the 8 MG, we obtained 812,974 effective reads (101,622 reads per sample), representing a total of 1977 ASVs. There were 1455 common ASVs between the two groups of samples.

### Alpha-diversity of intestinal microbiota in MN and MG

**Figure 1:**
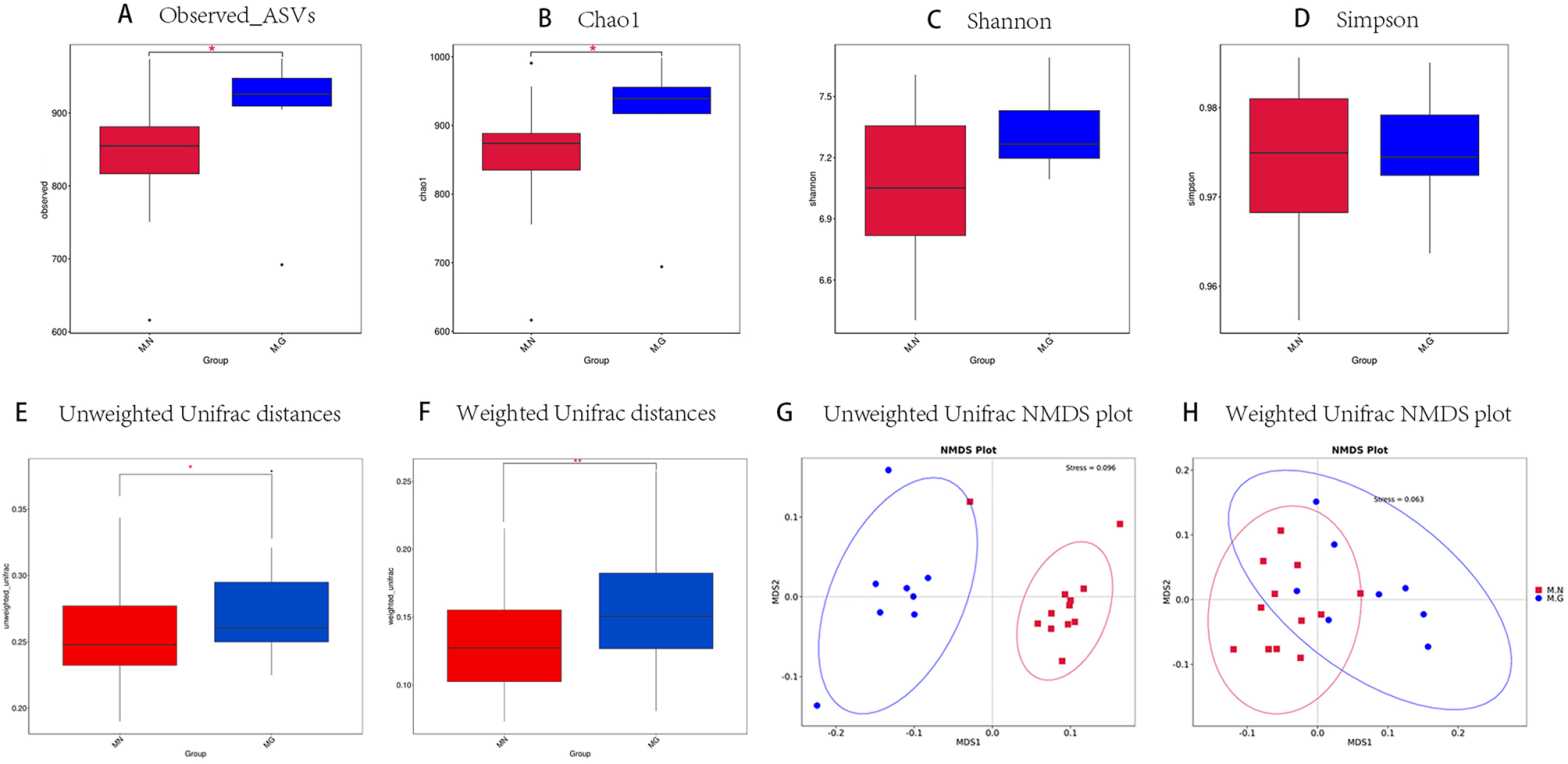
Differences in intestinal microbiota alpha-diversity and beta-diversity between MN and MG. (**A**) Differences in Observed_ASVs between groups. (**B**) Differences in Chao1 between groups. (**C**) Differences in Shannon between groups. (**D**) Differences in Simpson between groups. (**E**) Beta-diversity differences in unweighted Unifrac distances between groups. (**F**) Beta-diversity differences in weighted Unifrac distances between groups. (**G**) NMDS plot based on unweighted Unifrac distances of the two groups. (**H**) NMDS plot based on weighted Unifrac distances of the two groups. *M.G* rhesus macaques with liver fibrosis, *M.N* normal rhesus macaques, *Stress* less than 0.2 were considered NMDS can accurately reflect the degree of difference between samples. *P < 0.05, **P < 0.01, ***P < 0.001.

To analyze the diversity of the microbial community between the intestinal microbiota of MN and MG, we examined the alpha-diversity among the microorganisms in each sample. The Rarefaction curve for each index of alpha-diversity is depicted in Fig. S1A–G, and the curves tend to be flat for all samples, indicating reasonable sequencing data quantity. The alpha-diversity between the two groups is illustrated in Fig. 1A–D and Fig. S1H–J. It was apparent that the differences in Observed_ASVs and Chao1 between the intestinal microbiota of MG and MN were statistically significant in Fig. 1A,B (*P* = 0.047, *P* = 0.031). However, the differences in Shannon, Simpson, Dominance, Pielou_e, and Goods-coverage were not statistically significant (*P* = 0.16, *P* = 0.91, *P* = 0.91, *P* = 0.18, *P* = 0.85). It suggested that both MN and MG exhibited high diversity and good consistency in the composition of their intestinal microbiota. Furthermore, the total number of species in the intestinal microbiota of MG was greater than that of MN.

### Beta-diversity of intestinal microbiota in MN and MG

Alpha-diversity analysis revealed that MG had a greater total number of species in their intestinal microbiota compared to MN. To further investigate the compositional differences between the two groups, we utilized the Unifrac distance method to calculate inter-sample distances based on evolutionary information among microbial sequences in each sample. After obtaining a distance matrix, the Unifrac distance (specifically unweighted Unifrac) was further constructed using the abundance information of ASVs^18^. A larger Unifrac distance indicated a greater difference in species composition between samples. We calculated both weighted and unweighted Unifrac distances and assessed the differences in beta-diversity. As shown in Fig. 1E,F, without considering the sequence abundance, the composition of the intestinal microbiota in MN was more similar to that in MG (*P* = 0.02). However, when taking into account microbiota sequence abundance, the differences within each group decreased, while the statistical difference in similarity became more apparent (*P* = 0.007). Anosim analysis^19^ is a nonparametric test used to test whether the difference between groups is significantly greater than the difference within groups. An *R*-value greater than 0 means a substantial difference between the groups. If the corresponding *P*-value is less than 0.05, it indicates that the *R*-value is significantly greater than 0, thereby lending credibility to the result. The result of anosim analysis showed the unweighted Unifrac distances (*R* = 0.858156, *P* = 0.005) and weighted Unifrac distances (*R* = 0.392066, *P* = 0.005). Permutational MANOVA (PERMANOVA) can analyze the degree to which different grouping factors explain the variation observed in the samples. A higher *R*^2^-value indicates that the grouping scheme can better explains the difference between groups, and *P*-value less than 0.05 indicates a high level of confidence in the test. The result of PERMANOVA showed the unweighted Unifrac distances (*R*^*2*^ = 0.284846, *P* = 0.001) and weighted Unifrac distances (*R*^*2*^ = 0.245282, *P* = 0.001). The two types of analysis suggested a significant difference in the structure of the intestinal microbiota between MN and MG. The Non-Metric Multi-Dimensional Scaling (NMDS) non-linear analysis model can better reflect the differences in ecological data between samples^20^. Stress value less than 0.2 were considered NMDS can accurately reflect the degree of difference between samples, and the results of NMDS analysis (Fig. 1G,H) showed that after weighted, most of the microbiota components were the same between the intestinal microbiota of normal rhesus macaques and rhesus macaques with liver fibrosis. However, there were significant differences.

### Composition of phylum and genera in the intestinal microbiota from MN and MG

**Figure 2:**
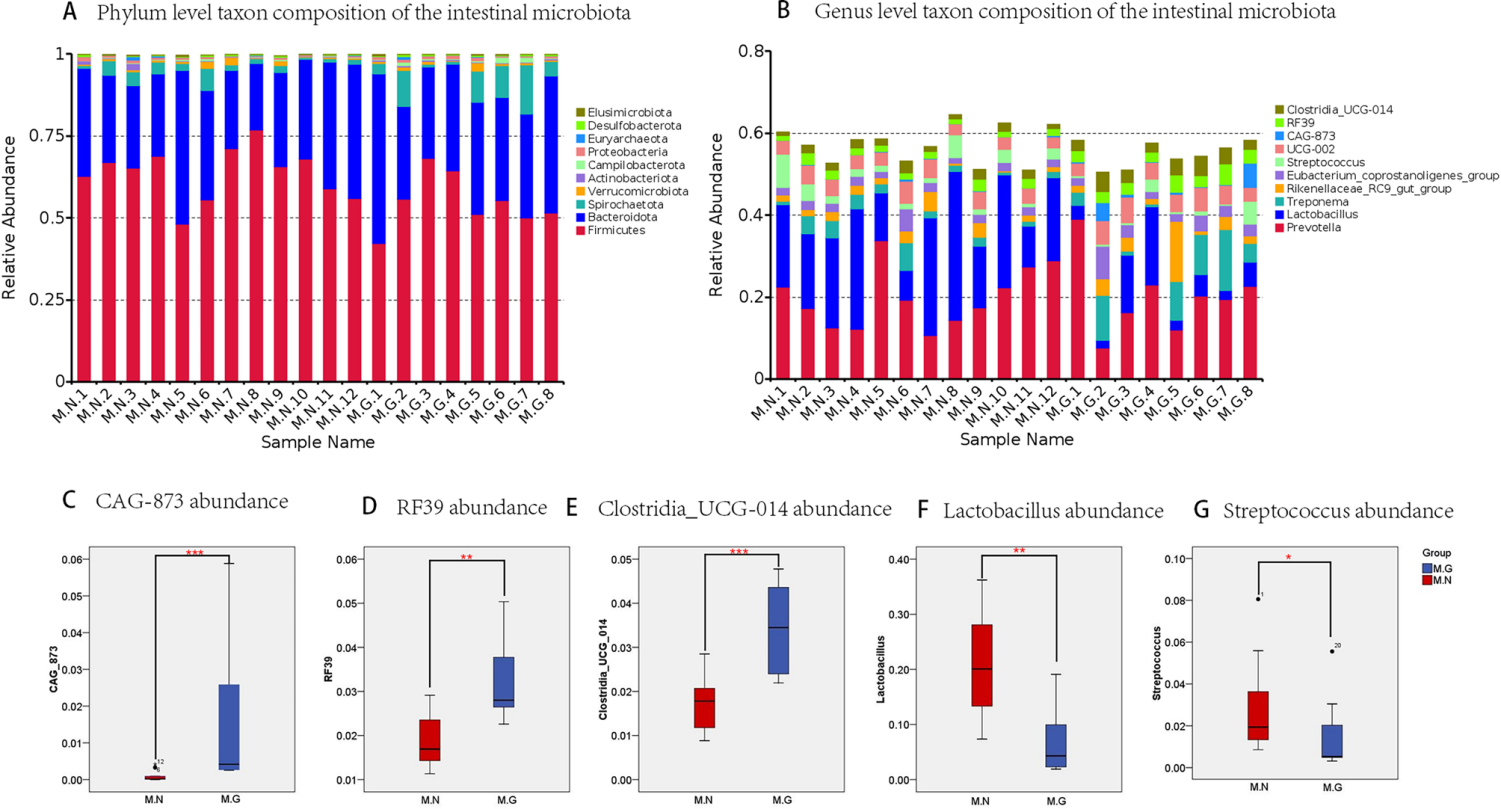
Composition differences at phylum and genus levels and specific abundance variations within the intestinal microbiota of MN and MG. (**A**) Top 10 phylum level taxon composition. (**B**) Top 10 genus level taxon composition. (**C**) Difference of *CAG-873* abundance between groups. (**D**) Difference of *RF39* abundance between groups. (**E**) Difference of *Clostridia_UCG-014* abundance between groups. (**F**) Difference of *Lactobacillus* abundance between groups. (**G**) Difference of *Streptococcus* abundance between groups. *M.G* rhesus macaques with liver fibrosis, *M.N* normal rhesus macaques. *P < 0.05, **P < 0.01, ***P < 0.001 level.

As shown in Fig. 2A, the dominant microbiota in both MN and MG were Firmicutes, Bacteroidota, and Spirochaetota, collectively accounting for over 85% of all intestinal microbiota. However, Metastat analysis was conducted at the phylum level using R software (Version 3.5.3) to assess significant differences between the two groups. MG had significantly more Campilobacterota (*P* = 0.02) compared to MN, whereas Firmicutes was significantly lower in MN (*P* = 0.03). As shown in Fig. 2B, the dominant genera in the intestinal microbiota of MG and MN were *Prevotella*, *Lactobacillus*, *Treponema*, *Rikenellaceae_RC9_gut_group*, and *Eubacterium_coprostanoligenes_group*. Notably, Metastat analysis demonstrated the intestinal microbiota of MG had significantly higher levels of *CAG-873*, *RF39*, and *Clostridia_UCG-014* compared to those of MN (Fig. 2C–E) (*P* < 0.001, *P* = 0.002, *P* < 0.001). Conversely, *Lactobacillus* and *Streptococcus* showed significantly lower levels in MG than in MN (Fig. 2F,G) (*P* = 0.002, *P* = 0.01).

### LEfSe analysis of intestinal microbiota in MN and MG

**Figure 3:**
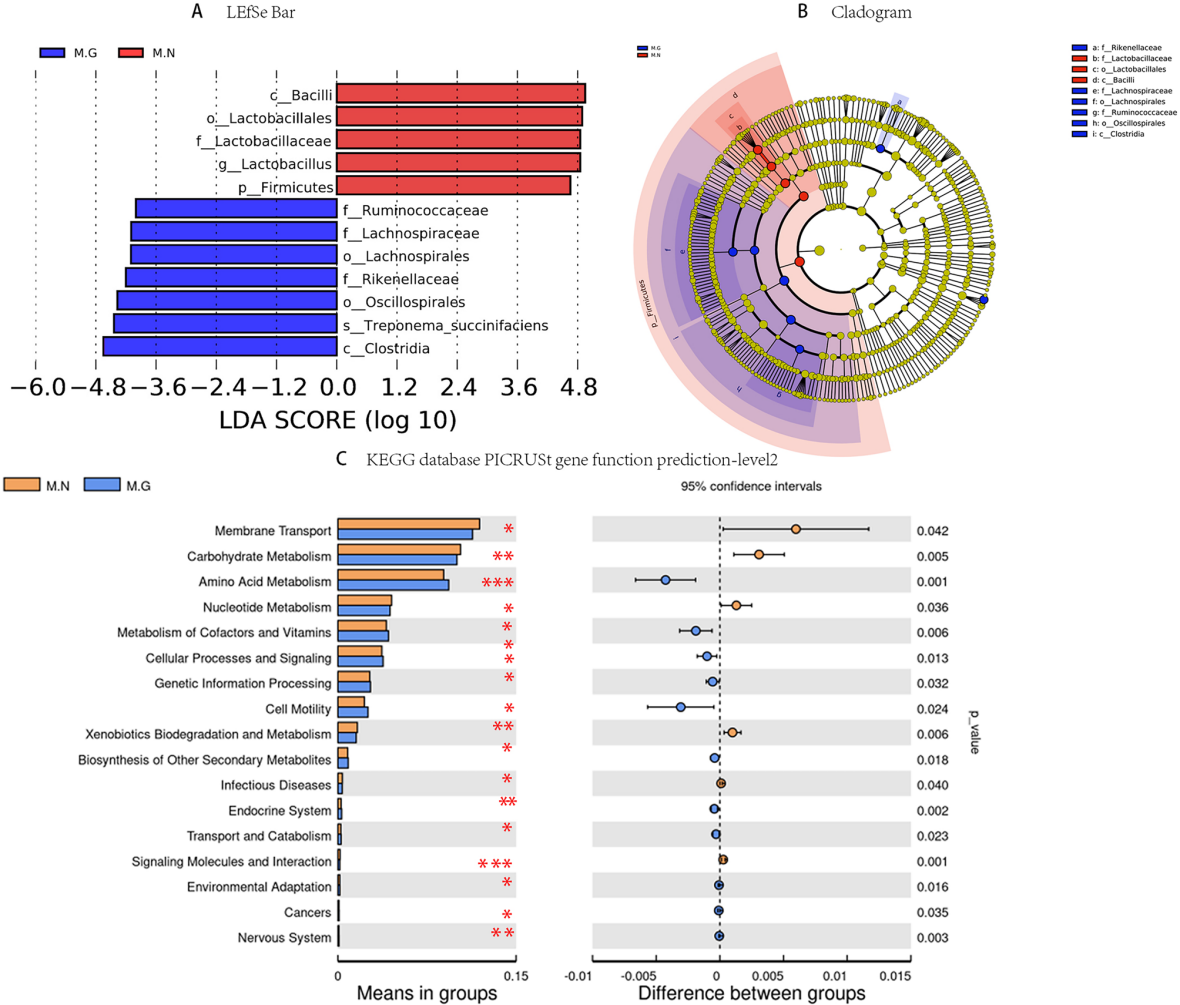
Insights from LEfSe analysis, taxonomic cladogram, and PICRUSt gene function prediction between MN and MG. (**A**) LDA scores of two groups obtained by LEfSe analysis (LDA scores ≥ 4). (**B**) Taxonomic cladogram generated from LEfSe analysis of 16S rRNA gene sequences. (**C**) PICRUSt gene function prediction in two groups on level 2 based on KEGG database. *M.G* rhesus macaques with liver fibrosis, *M.N* normal rhesus macaques. *P < 0.05, **P < 0.01, ***P < 0.001 level.

LEfSe^21^ is an analytical tool for the identification and interpretation of biomarkers in high-dimensional data. The algorithm can emphasize statistical significance and biological relevance between groups. In this study, we used the Linear Discriminant Analysis (LDA) score to determine the influence of differential bacterial species on intergroup differences. A higher LDA score reflects a greater contribution of the species to the observed intergroup differences. In this study, the LDA threshold was set at 4. As shown in Fig. 3A,B, the LEfSe analysis of the intestinal microbiota revealed 12 species exhibiting relative abundance differences between the two groups. Among these, the MG group contributed 7 species and the MN group contributed 5 species. The major microbiota responsible for distinguishing between the groups were *Bacilli*, *Clostridia* of the Firmicutes (*P* = 0.002, *P* = 0.001), and *Rikenellaceae* of the Bacteroidota (*P* = 0.04).

### Function prediction of PICRUSt in the MN and MG

**Figure 4:**
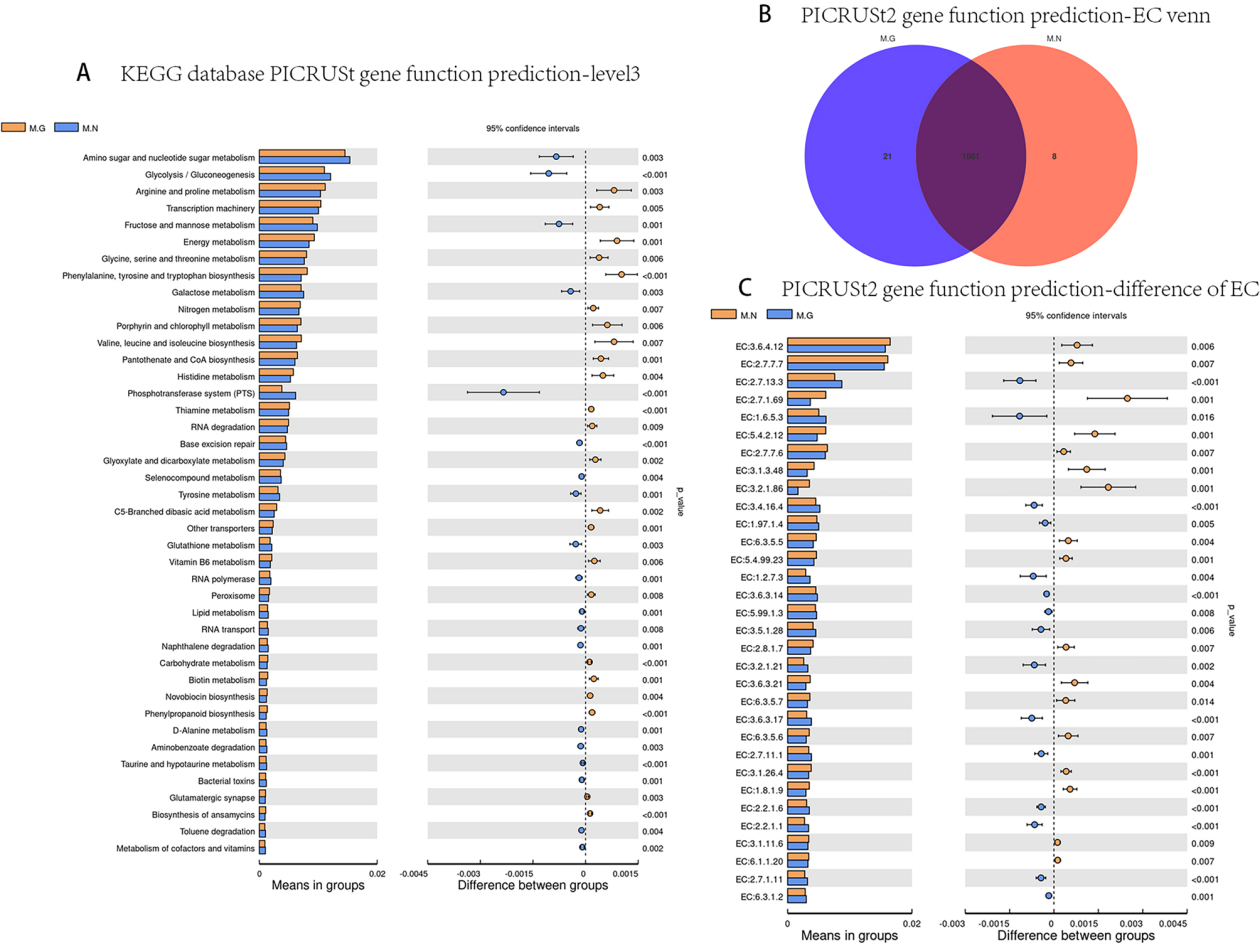
Detailed PICRUSt2 gene function prediction and EC database-based analysis between MN and MG. (**A**) PICRUSt gene function prediction of two groups on level 3 based on KEGG database. (**B**) Venn diagram of PICRUSt2 gene function prediction in two groups based on EC database. (**C**) Enzymes displaying significant differences between groups based on EC database. *M.G* rhesus macaques with liver fibrosis, *M.N* normal rhesus macaques.

PICRUSt is a bioinformatics package that enables the prediction of intestinal microbiota genome function using 16S rRNA. By employing PICRUSt function prediction based on the Kyoto Encyclopedia of Genes and Genomes (KEGG) database^22–24^, significant differences were observed in the functional composition abundance of intestinal microbiota between MN and MG, as depicted in Fig. 3C. The functional composition of the intestinal microbiota in MG and MN primarily exhibited enrichment in membrane transport within the environmental information processing pathway, as well as carbohydrate metabolism, amino acid metabolism, and nucleotide metabolism within the metabolism pathway. The degree of enrichment significantly differed between two groups. In Fig. 4A, the MN group displayed a higher abundance of genomes associated with amino sugar and nucleotide sugar metabolism, glycolysis metabolism, galactose metabolism within the carbohydrate metabolic pathway, and phosphotransferase system (PTS) genome within the membrane transport pathway. On the other hand, the MG group had more genomes related to arginine and proline metabolism, energy metabolism, phenylalanine/tyrosine and tryptophan biosynthesis, and pantothenate and CoA biosynthesis. Furthermore, PICRUSt2 function prediction based on the Enzyme nomenclature (EC) database within the KEGG database revealed differences in enzyme sequences within the intestinal microbiota of MN and MG, as shown in Fig. 4B. Enzymes enriched with significant differences (minimum abundance threshold of 0.003, *P* < 0.01) were analyzed between the two groups, and the results are presented in Fig. 4C. The MN group demonstrated a higher abundance of phosphotransferase (EC 2.7.7.7, EC 2.7.1.69), protein-tyrosine-phosphatase (EC 3.1.3.48), and 6-phospho-beta-glucosidase (EC 3.2.1.86). In contrast, MG exhibited a greater abundance of histidine kinase (EC 2.7.13.3), serine-type D-Ala-D-Ala carboxypeptidase (EC 3.4.16.4), and NADH: ubiquinone reductase (EC 1.6.5.3).

### Correlation analysis of the top 50 genera of the intestinal microbiota between MN and MG

**Figure 5:**
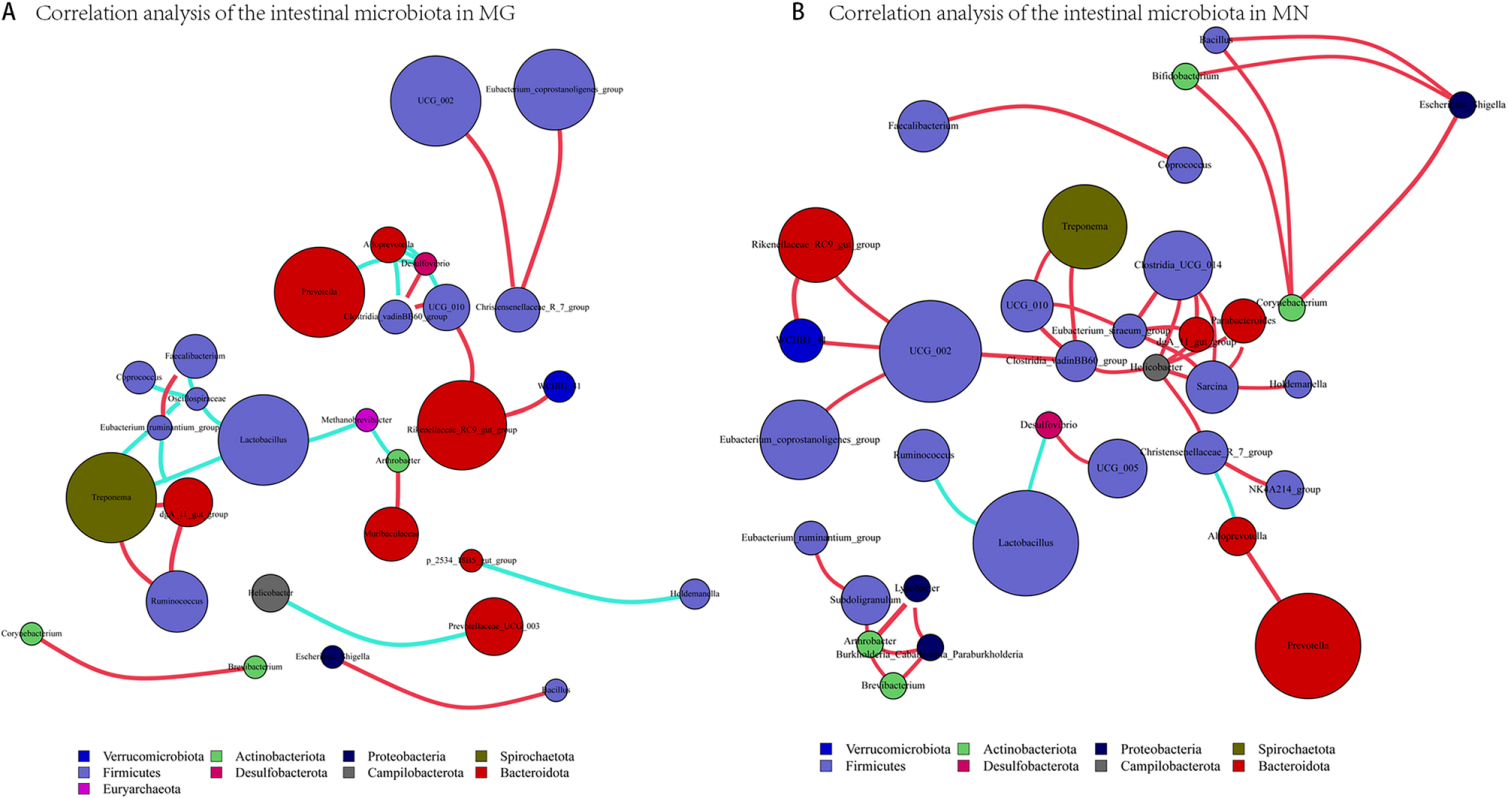
Correlation analysis of the top 50 genera in the intestinal microbiota of MN and MG. (**A**) Correlation analysis of the top 50 genera in the intestinal microbiota of MG. (**B**) Correlation analysis of the top 50 genera in the intestinal microbiota of MN. Selection basis for microbial genera: Spearman’s correlation coefficient greater than 0.7 or less than − 0.7 and *P*-value less than 0.01. Different nodes represent different genera, node size represents the average relative abundance of the genus, nodes of the same phylum are of the same color, and the color of the connecting lines between nodes corresponds positively or negatively to the correlation (red positive correlation, blue negative correlation).

Correlation analysis was performed by calculating Spearman’s correlation coefficient on the samples and filtering the matrix of species correlation coefficients to generate a correlation analysis network diagram. Figure 5A,B display the correlation analysis of the top 50 genera in the intestinal microbiota of MG and MN. The correlation coefficients were considered significant if they were greater than 0.7 or less than − 0.7, with a *P*-value less than 0.01. Compared to the MN group, the intestinal microbiota of MG showed changes in correlation patterns. *Prevotella* and *Alloprevotella* exhibited a shift from positive correlation to negative correlation, while *Lactobacillus* and *Treponema* transitioned from non-correlation to negative correlation. Additionally, *UCG_002* changed from positive correlation to non-correlation with *Rikenellaceae_RC9_gut_group*, *Eubacterium_coprostanoligenes_group*, and *WCHB1_41*. Overall, correlation among various intestinal microbiota of MG was reduced.

## Discussion

Rhesus macaques, a non-human primate species, have been widely used in recent years for researching human diseases. For instance, they have been used in cytomegalovirus model^25^ and Ebola virus model^26^, Alzheimer’s disease model^27^, liver disease model^28^, and type 2 diabetes model^29^. As the understanding of the mechanisms involving intestinal microbiota has advanced, scientists have discovered its association with the development of various diseases^30^. Currently, numerous studies^10,11^ have investigated the impact of intestinal microbiota on liver diseases through the gut-liver axis, thus opening up the possibility for treating liver diseases by modulating intestinal microbiota in vivo.

In this study, we examined the differences in the composition and functional composition of the intestinal microbiota between male MG and male MN using 16S rRNA gene sequencing. The results demonstrated statistically significant variations in both alpha-diversity and beta-diversity of the intestinal microbiota between MG and MN. Despite variations in composition and structure, the microbiota exhibited high diversity and good homogeneity between the two groups. These findings not only confirmed the efficacy of fecal sampling but also indicated that the intestinal microbiota in MG consistently underwent changes within the same dietary and housing environment.

In terms of intestinal microbiota composition, there were significant differences between MN and MG, both at the phylum level and the genus level. At the phylum level, the dominant microbiota common to both groups were Firmicutes, Bacteroidota, and Spirochaetota, accounting for about 85% or more of all intestinal microbiota, consistent with the findings of Cui et al.^12^. MG had significantly more Campilobacterota than MN, while the Firmicutes level was significantly lower, consistent with the findings of Mainz et al.^31^, who observed similar changes in a mouse model of alcoholic liver disease. Wang et al.^32^ also found that Firmicutes and Actinobacteriota were associated with changes in adiposity and leanness in pigs, suggesting a possible link between liver fibrosis development and fat and alcohol-metabolizing microbiota in vivo. At the genus level, the dominant genera in the intestinal microbiota of MG and MN were *Prevotella*, *Lactobacillus*, *Treponema*, *Rikenellaceae_RC9_gut_group*, and *Eubacterium_coprostanoligenes_group*. The study by Cui YF et al.^12^ also mentioned the high abundance of *Prevotella* in rhesus macaques. *Treponema* was another dominant microbiota observed in this study. However, it was discovered that beneficial bacteria like *Lactobacillus* were significantly lower in MG compared to MN. Liu et al.^33^ found that the probiotic *Lactobacillus rhamnosus GG* could prevent liver fibrosis in mice by inhibiting hepatic bile acid synthesis and enhancing bile acid excretion. Meanwhile, Yang et al.^34^ discovered that Metformin could reduce liver fibrosis in mice by enriching *Lactobacillus MF-1* in intestinal bacteria. Wang H et al.^16^ found that *Lactobacillales* and *Lactobacillus* significantly decreased in alcohol-induced chronic liver disease in rhesus macaques compared to normal rhesus macaques. These studies suggest that the occurrence of liver fibrosis leads to a decrease in the levels of beneficial bacteria like *Lactobacillus*. *Streptococcus* is an important intestinal microbiota, and most *Streptococcus* are non-pathogenic. However, pathogenic *Streptococcus* can cause bacterial infections. This study found that *Streptococcus* was reduced in MG. However, Jia et al.^35^ found human microbiota containing *Streptococci* encoding lantibiotic to be a potential risk factor for liver disease. Zhong et al.^36^ found that among patients with alcoholic fatty liver disease and alcoholic cirrhosis, *Streptococcus* enrichment was only found in the feces of patients with cirrhosis. Therefore, the number of *Streptococcus* may change dynamically with the progression of liver disease, and it may serve as a predictive microbiota for the progression of liver fibrosis to cirrhosis in the future. Furthermore, the levels of *CAG-87*3, *RF39*, and *Clostridia_UCG-014* were significantly higher in MG compared to MN. Wang et al.^37^ found that the abundance of intestinal microbiota *RF39* was reduced in a mouse model of alcohol use disorder, indicating its possible relation to alcohol metabolism in vivo. Qu et al.^38^ discovered a positive correlation between *Clostridia_UCG-014* and elevated bile acid levels in the feces of a chronic unpredictable mild stress mouse model, suggesting that its increased levels may be associated with liver fibrosis. There has been currently no literature reporting the association of *CAG-873* microbiota with liver disease, so further investigation is needed to determine its potential association with liver fibrosis.

Functional prediction of the intestinal microbiota using PICRUSt showed that the differences in the functional composition of the intestinal microbiota between MG and MN mainly focused on material metabolic pathways. MN had a higher abundance of amino sugar and nucleotide sugar metabolism, glycolysis metabolism, and PTS functions in their intestinal microbiota, while MG had a higher abundance of amino acid metabolism and energy metabolism functions. Wan et al.^39^ found that liver fibrosis in rats could alter the molecular structure of hepatic glycogen, affecting its metabolism and function. Xu et al.^40^ discovered that a novel Bmal1-IDH1/α-KG axis might regulate glycolysis in activated hepatic stellate cells, making it a potential therapeutic target for alleviating liver fibrosis. Jeckelmann et al.^41^ found that sugar PTS could mediate carbohydrate uptake and phosphorylation while regulating sugar carbon metabolism. Amino acids are crucial nutrients in the human body. This study revealed that after liver fibrosis, the intestinal microbiota’s ability to metabolize amino acids and generate energy increased. Results of PICRUSt2 function prediction showed that most of the enzymes with significant differences in abundance between the two groups belonged to the glucose metabolism and amino acid metabolism pathways. Hence, it can be hypothesized that liver metabolism is impaired after liver fibrosis, leading to alterations in the structure of glycogen molecules and decreased ability of the intestinal microbiota to metabolize sugars. This disruption of energy metabolism and amino acid metabolism further aggravates liver injury. Further research is needed to explore the relationship between the intestinal microbiota, various types of metabolic substances, and related mechanistic pathways after liver fibrosis.

Rhesus macaques are physiologically similar to humans and can provide a better simulation of the human intestinal system. Therefore, studying the relationship between the intestinal microbiota and liver fibrosis in rhesus macaques has advantages over studying patients with liver fibrosis. Rhesus macaques with liver fibrosis allow for easier control of diet, sample collection, and observation of the liver fibrosis process without treatment. In this study, there were no statistical differences in weight, age, and alanine transaminase (ALT) between MG and MN. However, aspartate aminotransferase (AST) levels were higher in MG, indicating the occurrence of liver injury in this group. Statistical differences in the composition of the flora between rhesus macaques with different degrees of liver fibrosis were not analyzed because the number of minimal fibrosis macaques and mild fibrosis macaques was less than three. Because all macaques were kept under the same conditions and there were no differences in age, body weight and liver function tests between the groups, we do not know the exact cause of liver fibrosis in the 8 rhesus macaques. Upon reviewing the birth records of these 8 MG, no rhesus macaques with the same parents were identified. Due to missing birth records of some MG parents, it is impossible to determine their familial relationships. In this study, we analyzed and discussed changes in the intestinal microbiota in MG. However, since the MG were obtained through screening and the exact etiology of the liver fibrosis is unknown at present, further investigation is required. Given the macaques’ susceptibility to diarrhea and the ineffectiveness of antibiotics, it is speculated that liver fibrosis may be related to refractory diarrhea, hepatobiliary diseases, and disorders of the intestinal microbiota. Additionally, rhesus macaques with moderate liver fibrosis may be affected by autoimmune liver disease, exacerbating the condition. To further investigate the mechanisms underlying the occurrence and development, all cases of liver fibrosis in rhesus macaques were left untreated in the present study. Future research is necessary to determine whether liver fibrosis is genetically linked, elucidate if its occurrence is associated with intestinal diseases or hepatobiliary diseases, and clarify whether liver fibrosis can be induced in MN through dietary modifications or chemical treatments. It is crucial for the identification of the exact cause of liver fibrosis and further exploration of the connection between liver fibrosis and changes in the intestinal microbiota. It is important to clinically validate the results of this study in the future due to physiological and dietary differences between rhesus macaques and humans.

## Conclusion

The intestinal microbiota of MG differed significantly from that of MN in terms of alpha-diversity, beta-diversity, dominant microbiota between groups, and functional prediction. The dominant genera of intestinal microbiota in both groups were *Prevotella*, *Lactobacillus*, *Treponema*. The levels of *Streptococcus* and *Lactobacillus* in MG were significantly lower than those in MN, while the levels of *CAG-873*, *RF39*, and *Clostridia_UCG-014* were significantly higher than those in MN. The above differential bacteria may primarily relate to liver fibrosis by affecting alcohol, bile acid, and fat metabolism. Functional prediction revealed that MG had more genes in glucose metabolism and substance transport pathways, whereas MN possessed more genes in amino acid metabolism and energy metabolism. Altered intestinal microbiota in MG may be associated with intestinal diseases or hepatobiliary metabolic disorder diseases. Since the subjects of this study were all male rhesus macaques, cause of liver fibrosis unclear, and no microbial metabolomics study was conducted, further studies should focus on the cause of liver fibrosis and the association between liver fibrosis and gene, relationships between intestinal microbiota and clinical indicators, as well as the correlation between intestinal microbiota and proteomics of liver fibrosis, providing guidance in exploring the mechanism of intestinal microbiota affecting liver fibrosis and its future potential use in treating liver fibrosis.

## Methods

### Animals

**Figure 6:**
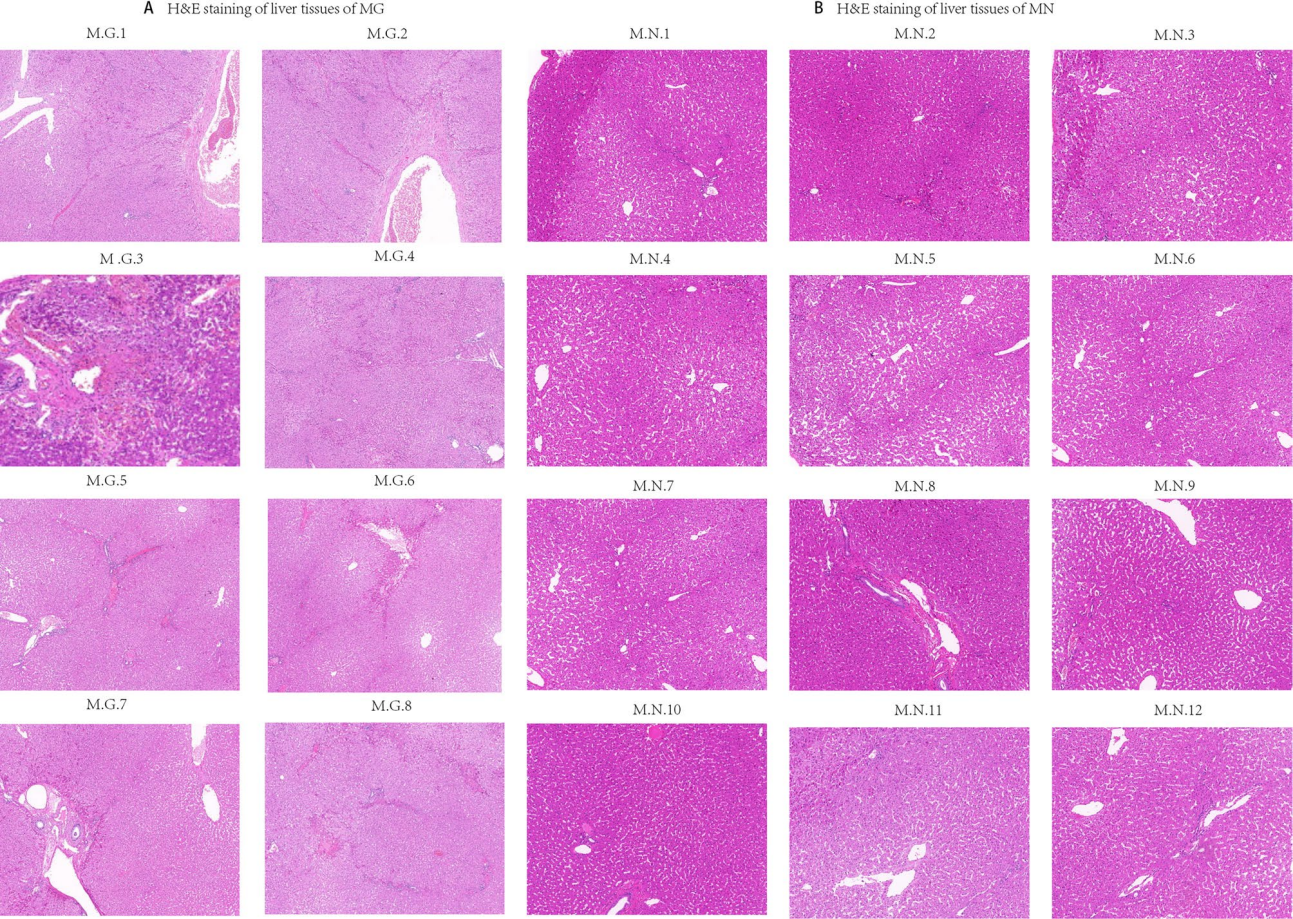
H&E staining of liver tissues fibrosis of MN and MG. (**A**) H&E staining of liver tissues of MG. (**B**) H&E staining of liver tissues of MN. *H&E* hematoxylin–eosin, *M.G.1–M.G.8* Rhesus macaques with liver fibrosis number, *M.N.1–M.N.12* normal rhesus macaques number.

Twenty male rhesus macaques (12 MN and 8 MG, 16–20 years old) were the second filial generation. Comparison of the specifics details between two groups are presented in Table 1, specific details concerning each macaque are presented in Table S1. All rhesus macaques were sourced from the Institute of Laboratory Animal Sciences, Sichuan Academy of Medical Sciences & Sichuan Provincial People’s Hospital and raised in captivity at the monkey base in Jianyang. All rhesus macaques were fed with the same diet (monkey growth and reproduction compound feedstuff produced by Beijing Keao Xieli Feed Co., Ltd. and apples provided by local fruit stores). 8 MG were incidentally discovered during the screening of macaques for liver transplantation experiments (following indications of liver abnormalities on abdominal ultrasound, shear wave elastography (SWE) was performed on one abnormal macaque, revealing liver fibrosis. Subsequently, liver puncture pathology biopsy confirmed the presence of 8 rhesus macaques with liver fibrosis. The results of hematoxylin–eosin (H&E) staining of puncture liver tissues from MG and MN are provided in Fig. 6. According to the Laennec histopathological grading scale, a total of 4 rhesus macaques with moderate liver fibrosis, 2 macaques with mild liver fibrosis and 2 macaques with minimal fibrosis, specific degree of liver fibrosis concerning each macaque are presented in Table S1. Puncture results were confirmed by Chunyou Lai, a liver doctor at the People’s Hospital of Sichuan Province, China. The collection and utilization of rhesus macaque samples were approved by the Animal Care and Use Committee of the Institute of Laboratory Animal Sciences, Sichuan Academy of Medical Sciences & Sichuan Provincial People’s Hospital (No.: 2023-004), and all experiments were performed in accordance with relevant guidelines and regulations. This study was in accordance with ARRIVE guidelines.

**Table 1:** Comparison of age, weight, and liver function test between rhesus macaques with liver fibrosis and normal rhesus macaques.

	Age (year)	Weight (kg)	ALT (U/L)	AST (U/L)
Rhesus macaques with liver fibrosis	17.89 ± 1.46	10.21 ± 1.45	54.53 ± 17.98	32.24 ± 8.45*
Normal rhesus macaques	18.08 ± 1.44	9.3 ± 1.81	57.67 ± 27.97	25.08 ± 8.14

Independent samples *t*-test was expressed with the mean ± standard deviation (SD).

*ALT* alanine transaminase, *AST* aspartate aminotransferase.

*P < 0.05.

### Sample collection

All rhesus macaques were fed the same diet, consisting of monkey growth and reproduction compound feedstuff produced by Beijing Keao Xieli Feed Co., Ltd, along with apples provided by local fruit stores. There were no recent gastrointestinal abnormalities or abnormal changes in body weight at the time of sample collection. A total of 12 MN samples and 8 MG samples were collected consistently. At approximately 8:00 a.m., the surface layer of fresh feces within the respective rearing rooms was collected using a sterile cotton swab, followed by gathering the central feces in a sterile PE tube also using a sterile cotton swab. All samples were promptly snap-frozen in a dry ice box upon collection and transported to the laboratory for preservation using dry ice. Subsequently, all samples were stored in a − 80 °C refrigerator.

### DNA extraction and detection

The total DNA was extracted from fecal samples using a magnetic bead method soil and fecal genomic DNA extraction kit (TianGen, China, Catalog#: DP712), following the provided instructions. The purity and concentration of DNA were assessed using 1% agarose gel electrophoresis to ensure that the extracted DNA was suitable for amplification.

### Polymerase chain reaction (PCR) and sequencing

For each collected sample, the 16S rRNA gene was amplified by PCR using universal bacterial primers 515F (5′-GTGCCAGCMGCCGCGGTAA-3′) and 806R (5′-GGACTACHVGGGTWTCTAAT-3′). The PCR products were evaluated via 2% agarose gel electrophoresis, confirming correct band sizes and meeting the requirements for library construction. The recovered product underwent purification using a universal DNA purification and recovery kit (TianGen). This gene then underwent high-throughput 16S rRNA sequencing at Novogene Co., Ltd. (Beijing, China). Library construction utilized the NEB Next® Ultra™ II FS DNA PCR-free Library Prep Kit (New England Biolabs, USA, Catalog #: E7430L), and the constructed libraries were qualified through Qubit and real-time fluorescent PCR quantification. Intestinal microbiota diversity was identified by PE 250 sequencing on the NovaSeq 6000 platform.

### Bioinformatic analysis

The reads from each sample were merged using FLASH software (Version 1.2.11) to obtain raw Reads data^42^, which were further processed using fastp software (Version 0.23.1) to acquire high-quality Clean Reads^43^. These sequences were matched with the Silva database, and chimeric sequences were removed to obtain Effective Reads^44^. ASVs were obtained using the DADA2 module of QIIME2 software (Version QIIME2-202006)^45^, then filtered to exclude ASVs with an abundance of less than 5^46^. Species annotation was performed using Mothur software with the Silva 138.1 database. ASVs was normalized using a standard of sequence number corresponding to the sample with the least sequences. The normalized ASVs were used to analyze microbial community diversity (alpha-diversity)^47^ and microbial community composition (beta-diversity)^18^. LEfSe analysis (LDA score threshold: 4) was conducted to identify biomarkers, and functional predictions were performed using PICRUSt2 software (Version 2.1.2-b) based on the KEGG database^48^.

### Statistical analysis

To compare the differences in intestinal microbiota between MN and MG. The indexes of alpha-diversity were statistically analyzed using Mann–Whitney U test. The indexes of beta-diversity were statistically analyzed using the Mann–Whitney U test, Anosim, PERMANOVA, and NMDS. The phylum and genus composition of intestinal microbiota was analyzed using the Metastat (Mann–Whitney U test). LEfSe analysis was carried out using the Mann–Whitney U test. Function prediction of PICRUSt and PICRUSt2 was performed using the Student’s unpaired *t*-test. Comparison of age, weight, and liver function was achieved by the independent Samples *t*-test. Values of *P < 0.05, **P < 0.01, and ***P < 0.001 were considered statistically significant for data analyses.

### Ethics statement

The study was in accordance with ARRIVE guidelines. Its protocol was approved by the Animal Care and Use Committee of the Institute of Laboratory Animal Sciences, Sichuan Academy of Medical Sciences & Sichuan Provincial People’s Hospital (No.: 2023-004). All methods were carried out in accordance with relevant guidelines and regulations.

### Supplementary Information

Supplementary Information.

## Data Availability

Intestinal microbiome sequencing data of normal and liver fibrotic rhesus macaques are available from the National Center for Biotechnology Information (NCBI) under accession number PRJNA987959, whole data can be found below: https://dataview.ncbi.nlm.nih.gov/object/PRJNA987959?reviewer=5qi4sq55h689406d98nqa79bca.
