# Supplementary material for: Metagenomic comparison of intestinal microbiota between normal and liver fibrotic rhesus macaques (Macaca mulatta)
Source: Sci Rep. 2024 Jul 8;14:15677. doi: 10.1038/s41598-024-64397-7 (PMC11231266; doi:10.1038/s41598-024-64397-7)
Supplement: Supplementary file 1 — Supplementary Information. [file 41598_2024_64397_MOESM1_ESM.docx]

**Metagenomic comparison of intestinal microbiota between normal and liver fibrotic rhesus macaques (Macaca mulatta)**

**Yuankui Wei1†, Junhui Li2†, Baoqiang Zhu1†, Qi Hu3, Ming Lan2, Jia Zhou2, Jianbo Luo2, Wanlong Zhu4, Yong Lai1, Enwu Long1,2,5*, Liang Zhou2***

1. **Figure S1**

**
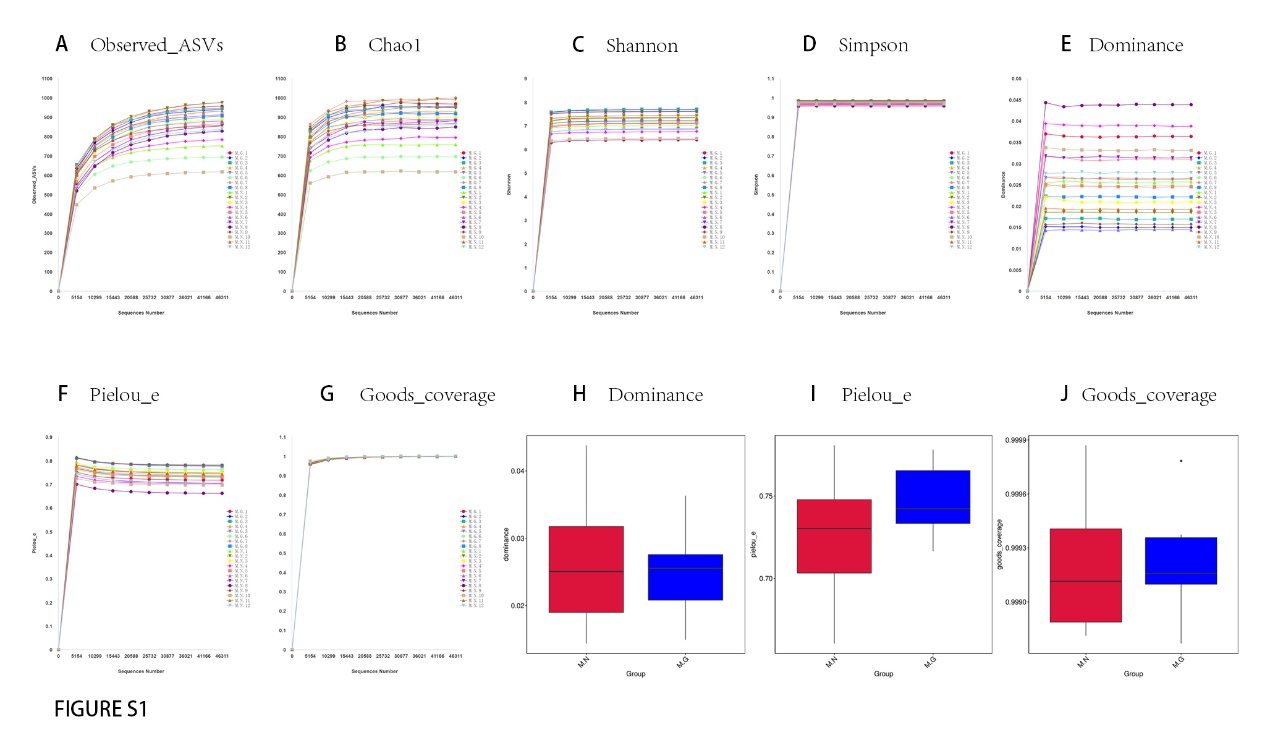
**

1. **Figure legend**

**FIGURE S1│** Rarefaction curves of alpha-diversity and differences in intestinal microbiota alpha-diversity between MN and MG. **(A)** Rarefaction curve of Observed_ASVs between two groups. **(B)** Rarefaction curve of Chao1 between two groups. **(C)** Rarefaction curve of Shannon between two groups. **(D)** Rarefaction curve of Simpson between two groups. **(E)** Rarefaction curve of Dominance between two groups. **(F)** Rarefaction curve of Pielou_e between two groups. **(G)** Rarefaction curve of Goods_coverage between two groups. **(H)** Differences in Dominance between groups. **(I)** Differences in Pielou_e between groups. **(J)** Differences in Goods_coverage between groups. **M.G**: Rhesus macaques with liver fibrosis. **M.N**: Normal rhesus macaques.

1. **Table**

**Table S1** Detailed data on age, weight, liver function tests and laennec system scores for each rhesus macaque.

| Number of rhesus macaques | Age (year) | Weight (kg) | ALT (U/L) | AST (U/L) | Laennec system scores |
| --- | --- | --- | --- | --- | --- |
| M.G.1 | 16 | 8.3 | 23.2 | 21.9 | 3 |
| M.G.2 | 19 | 9.8 | 42.3 | 27.0 | 3 |
| M.G.3 | 16 | 9.5 | 49.4 | 24.3 | 3 |
| M.G.4 | 18 | 13.0 | 50.3 | 34.1 | 1 |
| M.G.5 | 19 | 10.1 | 52.3 | 36.8 | 1 |
| M.G.6 | 18 | 10.6 | 66.7 | 45.9 | 2 |
| M.G.7 | 20 | 11.3 | 74.3 | 40.5 | 2 |
| M.G.8 | 17 | 9.1 | 77.8 | 27.4 | 3 |
| M.N.1 | 18 | 9.5 | 32.2 | 17.5 | 0 |
| M.N.2 | 18 | 8.6 | 109.2 | 36.8 | 0 |
| M.N.3 | 20 | 10.3 | 79.2 | 27.7 | 0 |
| M.N.4 | 16 | 9.1 | 39.4 | 20.0 | 0 |
| M.N.5 | 18 | 9.3 | 39.8 | 21.4 | 0 |
| M.N.6 | 17 | 11.3 | 36.3 | 17.3 | 0 |
| M.N.7 | 17 | 7.6 | 84.1 | 21.1 | 0 |
| M.N.8 | 20 | 6.6 | 86.3 | 23.2 | 0 |
| M.N.9 | 19 | 7.3 | 26.3 | 15.6 | 0 |
| M.N.10 | 16 | 8.1 | 42.3 | 26.7 | 0 |
| M.N.11 | 18 | 12.6 | 80.7 | 35.9 | 0 |
| M.N.12 | 20 | 11.3 | 36.3 | 37.8 | 0 |

Note: Numbers 1-8 are rhesus macaques with liver fibrosis, numbers 9-20 are normal rhesus macaques. ALT: Alanine Transaminase; AST: Aspartate Aminotransferase; Laennec system scores: 0 means no definite fibrosis, 1 means minimal fibrosis; 2 means mild fibrosis, 3 means moderate fibrosis; Independent Samples *t*-test were expressed with the mean ± standard deviation (SD). *p < 0.05, **p < 0.01, and ***p < 0.001.
